# Association of metformin use with Alzheimer’s disease in patients with newly diagnosed type 2 diabetes: a population-based nested case–control study

**DOI:** 10.1038/s41598-021-03406-5

**Published:** 2021-12-15

**Authors:** Junghee Ha, Dong-Woo Choi, Kwang Joon Kim, So Yeon Cho, Hyunjeong Kim, Keun You Kim, Youngseung Koh, Chung Mo Nam, Eosu Kim

**Affiliations:** 1 Department of Psychiatry, Institute of Behavioral Science in Medicine, Yonsei University College of Medicine, 50-1 Yonsei-ro, Seodaemun-gu, Seoul, 03722 Republic of Korea; 2 Cancer Big Data Center, National Cancer Control Institute, National Cancer Center, Gyeonggi-do, Republic of Korea; 3 Division of Geriatrics, Department of Internal Medicine, Yonsei University College of Medicine, Seoul, Republic of Korea; 4 Brain Korea 21 FOUR Project for Medical Science, Yonsei University College of Medicine, Seoul, Republic of Korea; 5 Department of Medicine, Yonsei University College of Medicine, Seoul, Republic of Korea; 6 Department of Preventive Medicine, Yonsei University College of Medicine, 50-1 Yonsei-ro, Seodaemun-gu, Seoul, 03722 Republic of Korea

**Keywords:** Alzheimer's disease, Clinical pharmacology

## Abstract

Metformin reduces insulin resistance, which constitutes a pathophysiological connection of diabetes with Alzheimer’s disease (AD), but the evidence of metformin on AD development was still insufficient and conflicting. We investigated AD risk in patients with newly diagnosed type 2 DM treated with metformin. This retrospective, observational, nested case–control study included patients with newly diagnosed type 2 DM obtained from the Korean National Health Insurance Service DM cohort (2002–2017). Among 70,499 dementia-free DM patients, 1675 AD cases were matched to 8375 controls for age, sex, and DM onset and duration. The association between AD and metformin was analyzed by multivariable regression analyses, adjusted for comorbidities and cardiometabolic risk profile. Metformin use was associated with an increased odds of AD (adjusted odds ratio [AOR] 1.50; 95% CI 1.23–1.83). The risk of AD was higher in patients with a longer DM duration. Furthermore, AD risk was significantly high in DM patients with depression (AOR 2.05; 95% CI 1.02–4.12). Given the large number of patients with DM who are taking metformin worldwide, a double-blinded, prospective study is required to determine the long-term cognitive safety of metformin.

## Introduction

The global prevalence of diabetes has increased significantly over the past few decades and is expected to be > 700 million by 2045^1^, with the majority of patients having type 2 diabetes mellitus (DM). Individuals with DM have a two-fold increased risk of Alzheimer’s disease (AD)^2^, and hyperglycemia itself is associated with impaired episodic memory and hippocampal atrophy, both characteristic signs of AD^3,4^. Understanding of the shared pathogenic mechanisms between DM and AD, such as insulin resistance, has increased the interest in the repurposing of antidiabetic drugs for the treatment of AD^5^.

Metformin is a first-line drug for DM treatment and is used by ≥ 120 million people worldwide^6^. Metformin can potentially be used to alleviate AD pathology because it can cross the blood–brain barrier and has a potent insulin-sensitizing property^7^. Studies have shown the beneficial effects of metformin on cognition are mediated through attenuation of insulin resistance and reduction of oxidative stress^8,9^. However, the speculation that metformin may play a protective role in AD pathogenesis has been challenged by several longitudinal studies. One study in the UK showed that long-term metformin use is associated with an increased risk of AD^10^, and an Australian one found that metformin-induced vitamin B_12_ deficiency was related to impaired cognitive performance in DM patients^11^. A population-based cohort study in Taiwan showed that metformin exposure in type 2 DM patients may be a risk factor for neurodegenerative diseases, including dementia and Parkinson’s disease^12^. Given the number of DM patients treated with metformin, the implications of these findings would have a massive impact on public health.

Despite the concerns generated from the abovementioned studies about safety pertaining to cognitive function in metformin users, a further sophisticated approach to control for potential confounding factors to reach a confirmatory conclusion on this issue is lacking. In earlier studies, DM patients accounted for approximately only 8% of the study population^10^ or there was no information on the severity and duration of DM, both directly associated with metformin dosage and length of administration^10,11^. Thus, it could not be conclusively established that metformin use, and not DM duration/severity, showed a significant relationship with an increased risk of AD. Moreover, the lack of validation of the AD diagnosis could be another critical issue^12^. As DM patients have an increased risk of vascular dementia, a potential ambiguity in the diagnostic classification may have distorted the results of the relationship between metformin use and AD risk. Therefore, in this study, we aimed to examine the effect of metformin use on the incidence of AD after adjusting for DM duration and severity using a nested case–control design. To address the possibility of diagnostic misclassification, we conducted a validation study, and to account for confounders by indication, we assessed cardiometabolic risk profile and prescription registry data of patients with newly diagnosed type 2 DM, assumed to be homogeneous in disease severity.

## Results

### Study population

**Figure 1:**
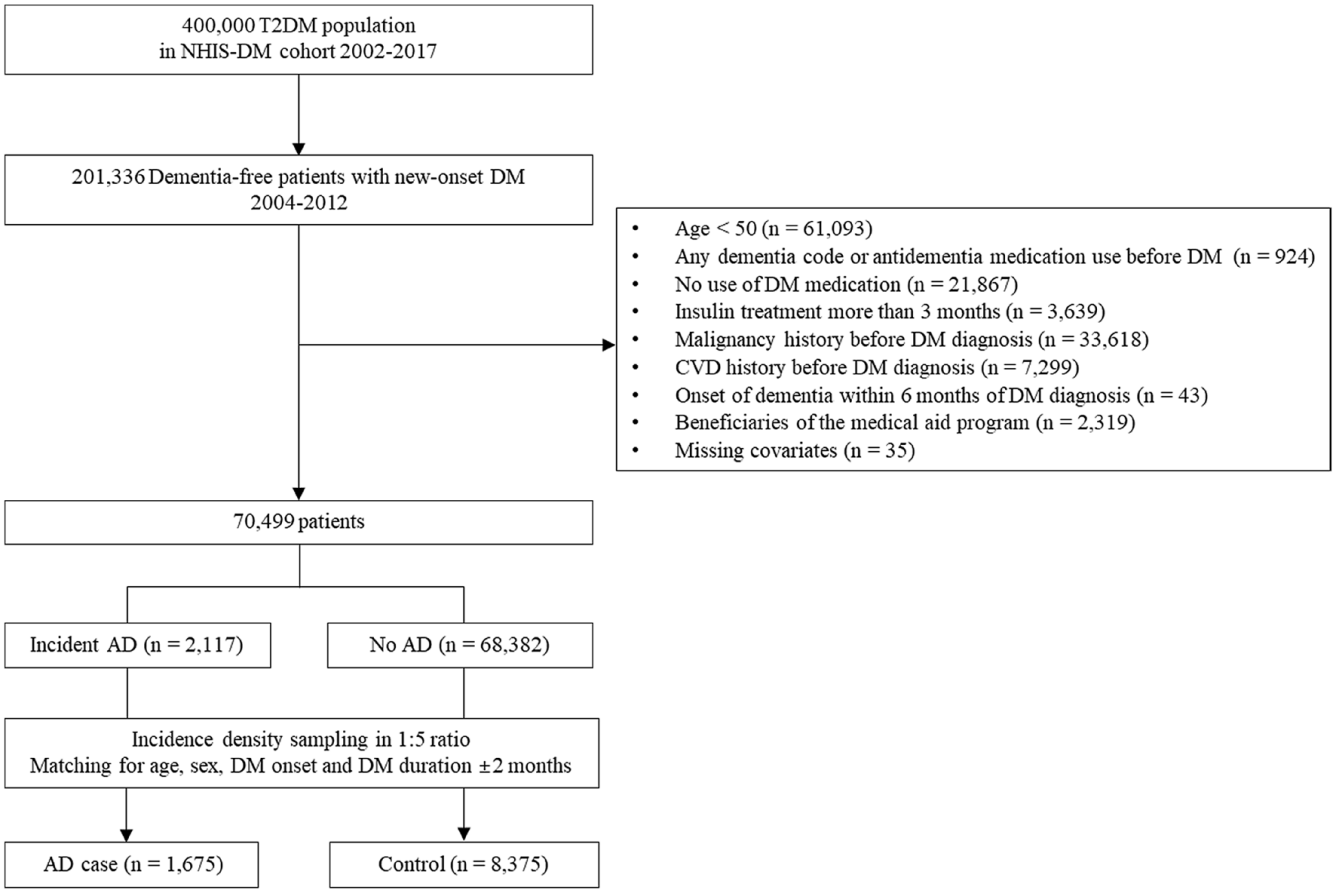
Flowchart of the study participant selection process. NHIS-DM, the Korean National Health Insurance Service-diabetes mellitus; CVD, cerebrovascular disease; DM, diabetes mellitus; AD, Alzheimer’s disease.

A total of 70,499 newly diagnosed type 2 DM patient, dementia-free, and aged ≥ 50 years were included. Of these patients, we identified 1675 AD cases and matched them with 8375 controls (Fig. 1). Table 1 shows the baseline characteristics of the cases and controls. Age, sex, date of DM onset, and DM duration did not differ between cases and controls after incidence density sampling. Compared with controls, cases were more likely to have depression (27.6% vs. 16.7%), and to use antiplatelet agents and insulin. The proportion of individuals with current smoking status and heavy alcohol intake was higher among cases than controls. The cases were less likely to physically active (63.3% vs. 69.2%).

**Table 1:** Study population characteristics of patients with Alzheimer's disease (cases) and controls.

Variables	Cases (n = 1675)	Controls (n = 8375)	*SMD*
	n (%)	n (%)	
Age			< 0.001
< 75 years	1074 (64.1)	5370 (64.1)	
≥ 75 years	601 (35.9)	3005 (35.9)	
Women	944 (56.4)	4720 (56.4)	< 0.001
Diabetes duration			0.002
< 5 years	194 (11.6)	969 (11.6)	
5–10 years	906 (54.1)	4524 (54.0)	
≥ 10 years	575 (34.3)	2882 (34.4)	
BMI			0.095
< 18.5 kg/m^2^	26 (1.6)	96 (1.1)	
18.5–22.9 kg/m^2^	406 (24.7)	1784 (21.3)	
23–25 kg/m^2^	416 (25.4)	2151 (25.7)	
≥ 25 kg/m^2^	793 (48.3)	4344 (51.9)	
Fasting blood glucose (mg/dL)^a^	134.10 ± 52.09	133.93 ± 49.55	0.003
BP (mmHg)^a^			
Systolic	133.36 ± 17.34	134.53 ± 17.48	0.068
Diastolic	80.53 ± 10.63	80.85 ± 10.79	0.030
Total cholesterol (mg/dL)^a^	203.33 ± 41.24	204.66 ± 41.73	0.032
Creatinine (mg/dL)^a^	0.99 ± 0.83	1.00 ± 0.86	0.009
Hypertension	1462 (89.1)	7200 (86.0)	0.094
Ischemic heart disease	534 (31.9)	2475 (29.6)	0.050
Dyslipidemia	1074 (64.1)	5419 (64.7)	0.012
CCI			0.050
0	545 (32.5)	2920 (34.9)	
1	398 (23.8)	1900 (22.7)	
2	732 (43.7)	3555 (42.4)	
aDCSI			0.102
0	1427 (85.2)	7383 (88.2)	
1	188 (11.2)	687 (8.2)	
2	60 (3.6)	305 (3.6)	
Depression	462 (27.6)	1395 (16.7)	0.266
Medication			
Statin	1119 (66.8)	5609 (67.0)	0.004
Aspirin	1103 (65.9)	5449 (65.1)	0.017
Antiplatelet	426 (25.4)	1584 (18.9)	0.157
Anticoagulant	83 (5.0)	259 (3.1)	0.095
Antihypertensive agents	1382 (82.5)	6671 (79.7)	0.073
Antiarrhythmic agents	238 (14.2)	980 (11.7)	0.075
Antidiabetic medication			
Alpha-glucosidase inhibitors	186 (11.1)	817 (9.8)	0.044
DPP-IV inhibitors	606 (36.2)	2430 (29.0)	0.153
Insulin	764 (45.6)	2890 (34.5)	0.228
SGLT-2 inhibitors	17 (1.0)	93 (1.1)	0.009
Sulfonylurea	1422 (84.9)	6905 (82.4)	0.006
Thiazolidinedione	274 (16.4)	1392 (16.6)	0.007
Smoking			0.083
None	1235 (73.7)	6318 (75.4)	
Past	156 ( 9.3)	874 (11.6)	
Current	284 (17.1)	1183 (14.1)	
Alcohol use			0.068
Low	1321 (78.9)	6755 (80.7)	
Moderate	238 (14.2)	1175 (14.0)	
Heavy	116 (6.9)	445 (5.3)	
Physical activity			0.126
Yes (≥ 1 time per week)	1060 (63.3)	5799 (69.2)	

*BMI* body max index, *CCI* Charlson Comorbidity Index, *aDCSI* adapted diabetes complication severity index, *DPP-IV* dipeptidyl peptidase IV, *SGLT-2* Sodium glucose cotransporter 2, *SMD* Standardized mean difference.

^a^Mean and standard deviation (SD) of the continuous independent variables in this study.

### AD risk associated with metformin use

During the study period, 1542 patients with AD cases (92.0%) and 7379 controls (88.1%) had used metformin (Table 2). Metformin use was significantly associated with an increased risk of AD (AOR 1.50; 95% CI 1.23–1.83) after controlling for potential confounders. Although a dose–response relationship between metformin use and AD risk was not observed in the cDDD, dose-dependency was revealed in the cDDD per day, with moderate to high daily doses being the most threatening. The strongest association with AD risk occurred in metformin users with the highest cDDD per day (AOR 1.66; 95% Cl, 1.34–2.07).

**Table 2:** Risk of Alzheimer’s disease associated with metformin use in diabetes mellitus patients. Analysis was adjusted for the following covariates: hypertension, ischemic heart disease, dyslipidemia, Charlson Comorbidity Index, Diabetes Complications Severity Index, depression, statin use, aspirin use, antiplatelet use, anticoagulant use, antihypertensive drug use, antiarrhythmic drug use, use of antidiabetic medications, fasting blood glucose levels, systolic blood pressure, diastolic blood pressure, total cholesterol levels, creatinine levels, body mass index, smoking status, alcohol consumption, and physical activity.

	Cases (n = 1675)	Controls (n = 8375)	Crude OR (95% Cl)	AOR (95% Cl)
	n (%)	n (%)		
**Metformin use**				
Never-users	134 (8.0)	996 (11.9)	1.00	1.00
Users	1542 (92.0)	7379 (88.1)	1.57 (1.30–1.90)	1.50 (1.23–1.83)
**Cumulative dose of use**				
Never-user	134 (8.0)	996 (11.9)	1.00	1.00
Ever user				
Q1 (< 181cDDDs)	415 (24.8)	1818 (21.7)	1.70 (1.38–2.10)	1.66 (1.34–2.06)
Q2 (181–507 cDDDs)	374 (22.3)	1853 (22.1)	1.51 (1.22–1.87)	1.43 (1.15–1.79)
Q3 (508–1044 cDDDs)	392 (23.4)	1828 (21.9)	1.60 (1.29–1.97)	1.48 (1.19–1.85)
Q4 (≥ 1045 cDDDs)	360 (21.5)	1870 (22.3)	1.44 (1.16–1.79)	1.35 (1.08–1.70)
**Cumulative dose per day**				
Never-user	134 (8.0)	996 (11.9)	1.00	1.00
Ever user				
Q1 (< 0.25 cDDDs/day)	445 (26.6)	2520 (30.1)	1.32 (1.07–1.63)	1.37 (1.10–1.70)
Q2 (0.25–0.31 cDDDs/day)	268 (16.0)	1227 (14.7)	1.64 (1.31–2.05)	1.50 (1.19–1.90)
Q3 (0.32–0.46 cDDDs/day)	400 (23.9)	1830 (21.9)	1.65 (1.33–2.04)	1.52 (1.22–1.90)
Q4 (≥ 0.47 cDDDs/day)	428 (25.6)	1802 (21.5)	1.79 (1.45–2.21)	1.66 (1.34–2.07)

*cDDDs* cumulative defined daily doses, *AOR* adjusted odds ratio, *CI* confidence interval.

### Association of metformin use and AD risk stratified by DM duration and depression

**Figure 2:**
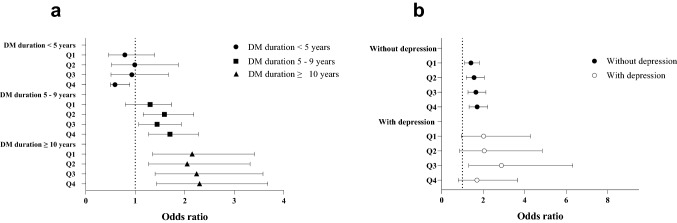
Association between metformin use and Alzheimer’s disease risk according to diabetes mellitus (DM) duration and depression. Adjusted odds ratios with 95% confidence intervals for multivariable models evaluating associations between metformin use and incident Alzheimer’s disease. (**A**) Risk of Alzheimer’s disease associated with metformin use according to DM duration (**B**) Risk of Alzheimer’s disease associated with metformin use in patients with and without depression. Reference is metformin never-users, and the average cDDD per day is presented as quartiles. cDDDs cumulative defined daily doses; AOR, adjusted odds ratio; CI, confidence interval. *Analysis was adjusted for the following covariates: hypertension, ischemic heart disease, dyslipidemia, Charlson Comorbidity Index, Diabetes Complications Severity Index, depression, statin use, aspirin use, antiplatelet use, anticoagulant use, antihypertensive drug use, antiarrhythmic drug use, use of antidiabetic medications, fasting blood glucose levels, systolic blood pressure, diastolic blood pressure, total cholesterol levels, creatinine levels, body mass index, smoking status, alcohol consumption, and physical activity.

To analyze the effect of metformin on AD according to DM duration, we stratified the DM duration into three categories: < 5 years, 5–9 years, and > 10 years. Metformin users consistently showed a higher risk of AD than metformin never-users except in people with DM duration < 5 years. No statistical significance was found in the people with DM duration < 5 years (AOR 0.88; 95% CI 0.54–1.43). AD risk was the highest in people with a DM duration > 10 years (AOR 1.48; 95% CI 1.14–1.91; for DM duration of 5–9 years: AOR 2.18; 95% CI 1.41–3.39; Fig. 2a; see Supplementary Table e1 online). These results suggest that metformin use increases the AD risk, with a synergistic effect of DM duration on AD risk. However, the effect of metformin on AD was not significant in people who had a shorter DM duration.

### Association of metformin use and AD according to depression

As depression is a risk factor or often precursor to AD, a higher prevalence of depression in the case population may act as a confounding factor in assessing the risk of AD in metformin users. Therefore, we performed subgroup analyses in prespecified strata of clinical interest to assess effect modification. The association with AD in metformin users was prominent in patients with and without depression (Fig. 2b). Of note, the risk of AD associated with metformin use was substantially greater in people with depression (AOR 2.05; 95% CI 1.02–4.12; Fig. 2b), while the significance of this association was maintained in patients without depression (1.57; 95% CI 1.24–1.98, see Supplementary Table e2 online). This suggests that the association between metformin and AD risk is independent of depression, although depression has an additive effect on AD risk.

### Subgroup analyses

To examine the effect of possible confounding factors on AD, we further assessed the demographic and clinical characteristics of metformin users and never-users; the differences in comorbidities and demographic characteristics are listed in Supplementary Table e3. Depression was more common among metformin users than among never-users (18.8% vs. 15.6%). Statin use were more prevalent among metformin users (65.8% vs. 60.6%). Severity of the comorbidity burden, indicated by a CCI score ≥ 2, was higher in metformin users than in never-users (43.2% vs. 38.1%). Fasting blood glucose levels were slightly higher in metformin users than in metformin never-users, although there was no significant difference between their aDCSI scores. However, the subgroup analyses defined by different comorbidity and medication uses did not disclose any significant alteration in the observed effect of metformin on AD, except in subjects without hypertension or in those with antiarrhythmic use (see Supplementary Figure e1 online).

### Sensitivity analyses

We further conducted nearest neighbor matching to control covariate imbalance. A total of 2027 patients with AD and 9,708 without AD were matched in a 1:5 ratio. The demographic characteristic of the two samples were almost similar (see Supplementary Table e4). Even after the matching, metformin users still exhibited a significantly higher risk of AD than metformin never-users (AOR 1.25, 95% CI 1.07–1.44; see Supplementary Table e5).

## Discussion

In a national longitudinal nested case–control study, we evaluated 1675 AD cases and 8375 controls matched by age, sex, time of DM onset, and DM duration. The principal findings were as follows: (1) metformin use was associated with a significantly increased AD risk; (2) the strength of the association increased with the cumulative daily defined dose per day in metformin users; (3) the association between metformin use and AD was the strongest in patients with a longer DM duration; and (4) the metformin-associated increase in AD risk was independent of the presence or absence of depression, although the risk was significantly higher in individuals with depression than in those without depression—this finding suggests that depression might enhance the association between metformin use and AD risk.

The association between metformin use and AD incidence has been controversial. Some studies have revealed that metformin use is associated with a lower risk of AD^13,14^ or has no association with AD risk^15^, whereas others, in agreement with our findings, have shown that metformin use is associated with an increased AD risk^10,12^. Indeed, previous epidemiological studies on metformin use and AD risk have shown substantial differences in study population and design. A population-based study conducted in Singapore showed that long-term metformin exposure reduces the risk of cognitive decline^14^; another study conducted in Taiwan reported that patients with DM treated with metformin had a lower risk of dementia than those who were not prescribed medication^16^. Several issues should be considered when interpreting the results of these earlier studies. In the study conducted in Singapore, the cognitive outcome was measured using only the Mini-Mental State Examination, which is does not indicate the diagnosis of dementia^14^. The diagnosis of dementia was indicated by “all-cause dementia,” which would also include dementias other than AD^16^. Further, the duration and severity of DM were not considered, although these factors are closely associated with the dose and duration of metformin treatment. Contrarily, in the latest study using reliable neuropsychological cognitive assessment tools (i.e., Repeatable Battery for Assessment of Neuropsychological Status and the Frontal Assessment Battery) reported cognitive dysfunction in community-dwelling elderly metformin users^17^.

Similarly, animal studies examining the effects of metformin on AD pathology have yielded conflicting results. Our finding that metformin users have a greater AD risk is consistent with that of animal studies, which have shown that metformin increases β-amyloid concentration by elevating the level of β-site amyloid precursor protein cleaving enzyme 1 or increases insoluble tau species in mice^18,19^. Contrastingly, other studies have demonstrated positive effects—metformin prevented β-amyloid generation by improving insulin resistance, inducing hippocampal neurogenesis, or reducing tau phosphorylation through the protein phosphatase 2A-dependent pathway^20^.

However, most of these observations were made in mouse cortical neurons, and the duration of metformin treatment was short when compared to DM treatment durations in human patients. A recent study showed sex-dependent dissociable effects of metformin on cognitive function. Female APP mice treated with metformin showed improved learning and cognitive function, whereas male mice showed a worsening memory function^21^. A longitudinal study found that metformin use was associated with greater decline in delayed memory among apolipoprotein E (APOE) ε4 carriers with AD, while the opposite was found in cognitively normal individuals^22^. Another animal study also revealed that the effect of metformin on the memory of aged mice may change in an APOE genotype-dependent manner^23^. These results suggest that the mechanisms underlying metformin use and cognition are complex and can generally be considered multifactorial.

Depression is a known important risk factor for dementia and cognitive decline among patients with DM^24^. We found that the prevalence of depression was higher in AD cases than in controls. Additionally, a history of depression was found more frequently in metformin users than in non-users. As depression is a major risk factor or a prodromal symptom of AD^25^, the higher prevalence of depression in the case population may confound the result. However, on further stratified analyses, we found that the metformin-induced increase in AD risk was independent of depression. Among metformin users, AD risk was higher in those with depression, consistent with a previous finding that depression can accelerate cognitive decline in patients with DM^26^.

The pathophysiological mechanism underlying the association between metformin use and AD risk has not been elucidated. One possible explanation is that long-term metformin use is associated with vitamin B_12_ deficiency, possibly leading to cognitive decline^11^. In a randomized controlled trial, vitamin B_12_ deficiency was reported in 4.3% of 859 participants who were metformin users, much higher than the proportion in the placebo group (2.3%)^27^. The complex role of 5' adenosine monophosphate-activated protein kinase (AMPK) could provide another explanation for the increased AD risk among metformin users. Metformin is known to exert its antidiabetic effect through AMPK activation, which plays multiple biological roles in cellular energy homeostasis, insulin signaling, and glucose metabolism^28,29^. Thus, metformin was expected to have a positive effect on cognitive function by activating AMPK^8^. However, there are controversial findings on the role of AMPK in the brain^30^, and opposing effects of AMPK activation on cognitive function have also been reported^31,32^. It was shown that AMPK overactivation increases neural apoptosis and neuronal networks dysfunction, whereas modest activation induces neurogenesis and improves cognitive function^33^. Recently, studies have shown that the abnormal AMPK α1 upregulation in postmortem human AD brain tissue plays an important role in mediating AD-related cognitive impairment and synaptic failure^34^. Given that the AMPK activation induced by metformin use affects mostly the activation of the α1 isoform^28^, it is possible that metformin may cause neuronal dysfunction by upregulating AMPK α1.

This study has several strengths. First, we used well-established and validated national longitudinal data, sourced from the NHIS, and included follow-up data on patients with type 2 DM from 2002 to 2017. These data provided sufficient details of lifestyle and clinical information to facilitate rigorous statistical adjustment. After matching for DM duration and time of DM onset, we could generate a comparable sample of control participants selected from the same population as that used for obtaining cases, thereby eliminating the guarantee-time bias. Second, we applied additional exclusion criteria to the operational AD definition, commonly used in current epidemiological studies^35^, and compared the diagnoses with actual hospital data for validation. Misclassification of dementia types may contribute to different outcomes, although few earlier studies have conducted a validation process for AD diagnosis. Third, we conducted subgroup analyses to observe the potential effects of various confounding factors, including dyslipidemia, other medication use, comorbidities, and depression, and our main findings remained robust. However, despite the study’s useful findings and strength, our study has some limitations that should be addressed. First, the NHIS claims database is potentially susceptible to measurement errors arising from coding inaccuracies. To minimize such errors, we defined patients with AD as those who visited a physician at least twice in a given year and were treated with anti-dementia medications; other diseases that could be mistaken for AD were strictly excluded. We then validated the accuracy of this definition using an independent sample from two hospitals, with a PPV of 83%. Second, the timepoint of the registration of the AD diagnostic code may not have coincided with the exact time of AD onset, leading to the possibility of a substantial gap between the time of diagnosis and disease onset. Third, the diagnostic bias of DM may be attributed to AD incidence as patients with DM visit hospitals more frequently. However, in this study, cases and controls were selected from the same DM population, wherein the DM duration was matched to minimize the selection bias. Fourth, this was an observational study, not a randomized trial, and, therefore, we should also consider the possibility of hidden bias between patients who received metformin and those who did not. In addition, there was insufficient information regarding the severity of diabetes (e.g., hemoglobin A1c level) from NHIS. Lastly, APOE and vitamin B_12_ levels were not evaluated. Recent studies have indicated that metformin use was associated with cognitive decline in patients with AD depending on the APOEε4 carrier status^22^, and that long-term metformin use is associated with vitamin B12 deficiency, which is related to cognitive impairment^11^. Therefore, other unmeasured factors may influence the relationship between metformin use and AD risk. Further studies in an independent cohort including these additional clinical variables and large-scale prospective study are necessary to conform the long-term safety of metformin use.

In conclusion, we found that metformin use was associated with higher AD incidence among patients with newly diagnosed DM. Additionally, increased AD risk associated with metformin use was more evident in patients with a longer DM duration and in those with depression. There is an increasing demand to identify modifiable risk factors for AD, as therapeutic interventions for AD have failed. The high global prevalence of DM and metformin use necessitates further experimental study to identify mechanisms that link metformin use with AD risk. In addition, larger prospective studies with more clinical information are required to obtain confirmatory results on the cognitive safety of metformin.

## Methods

### Study design and data source

We used a 2002–2017 data set from the Korean National Health Insurance Service (NHIS)-DM cohort. It contains data of 400,000 patients with type 2 DM which corresponds to a sample of approximately 23% of the entire type 2 DM population (ICD E11-14) in the 35–85 years age group in South Korea. This dataset included all inpatient and outpatient medical claims data, including data on prescription drug use, diagnostic and treatment codes, and primary and secondary diagnosis codes. It also included the National Health Screening Program (NHSP) data. Since 2000, the Korean government has implemented an obligatory NHIS, which covers up to 98% of the entire Korean population, and all insured adults are eligible for the NHSP, and recommended to undergo a standardized health check-up every 1–2 years. The Korean NHIS claims database records diagnoses based on the International Statistical Classification of Disease and Related Health Problems, Tenth Revision (ICD-10) codes. This study was approved by the Institutional Review Board of Yonsei University Health System (approval no. 4-2019-0674), who waived the requirement for informed consent because of the use of deidentified patients’ data. All analyses adhered to the guidelines and regulations of the ethics committee of Yonsei University Health System.

### Case selection and validation

From the Korean NHIS-DM cohort, a total of 201,336 dementia-free patients with newly diagnosed DM who had undergone a health check-up between 2004 and 2012 were enrolled, and follow-up data collected until December 2017 were reviewed. We excluded (1) patients < 50 years (n = 61,093); (2) patients diagnosed with dementia before the DM diagnosis (n = 924); (3) patients who had not used antidiabetic medications (n = 21,867); (4) patients receiving insulin treatment for > 3 months (n = 3639); (5) patients with a history of malignancy before the DM diagnosis (n = 33,618); (6) patients with a history of cerebrovascular disease (CVD) before the DM diagnosis (n = 7299); (7) patients with the onset of dementia within 6 months of DM diagnosis (n = 43); (8) beneficiaries of medical aid programs (n = 2319). Patients with a history of CVD and malignancy were excluded because stroke or vitamin deficiencies associated with these diseases might increase the risk of dementia and cognitive impairment. Finally, we enrolled 70,499 patients, including 2117 patients diagnosed with incident AD until 2017 (Fig. 1). The following ICD-10 codes were used to identify an AD case: F00 or G30 (AD), F01 (vascular dementia), F02 (dementia with other diseases classified elsewhere), and F03 (unspecified dementia). To focus on AD, attempts were made to increase the probability of including only well-defined AD cases. An eligible AD case involved an individual diagnosed based on the F00.0, F00.1, F00.2, or F00.9 code, followed by ≥ 2 events of prescriptions for an anti-dementia medication (rivastigmine, galantamine, memantine, or donepezil) within a year of the diagnosis. Individuals diagnosed with Parkinson’s disease, stroke, motor neuron disease, normal pressure hydrocephalus, or cancer before the diagnosis of dementia as well as those with any other specific dementias, such as vascular dementia, were excluded from the study population. The index date was defined as the date of AD diagnosis. This algorithm was a modified version of the case-identification procedure from an earlier study that used the NHIS data^35^. To evaluate the accuracy of the algorithm, a validation study was conducted in two teaching hospitals with 737 patients, and the positive predictive value (PPV) was 83%. For the main analysis, 1675 cases and 8375 controls were matched in a 1:5 ratio. Control participants were randomly selected from the DM cohort, matched to the cohort affected patients based on age, sex, time point of DM onset and DM duration.

### Exposure to metformin

Metformin use was defined as a total prescription of metformin for 60 > cumulative DDDs after DM treatment onset^36^. Exposure to metformin was assessed from the first prescription to the index date. We calculated cumulative defined daily dose (cDDD) according to the World Health Organization definition^37^ and described metformin exposure according to the following three criteria: (1) ever user; (2) cDDD; and (3) time-weighted mean (TWM) cDDD per day, i.e., the cumulative sum of metformin cDDDs in each patient was divided by the number of days that patient received metformin to produce the TWM cDDD of metformin in each 1-day period^38^, were classified by quartiles.

### Potential confounders

We obtained information on selected comorbid conditions from inpatient and outpatient hospital diagnoses. The existence of hypertension, ischemic heart disease, dyslipidemia, CVD, chronic kidney disease, depression, and prescription medication information prior to the index date. The Charlson Comorbidity Index (CCI) was measured during the year before the index date. Adapted Diabetes Complications Severity Index (aDCSI) was measured from DM diagnosis to the index date^39^. Fasting blood glucose levels, systolic blood pressure, diastolic blood pressure, total cholesterol levels, creatinine levels, BMI (< 18.5, 18.5–22.9, 23.0–25.0, and ≥ 25.0 kg/m^2^), smoking status (none, past, and current), alcohol consumption (low: < 1 time/week, moderate: 1–4 times/week, and heavy: 5–7 times/week), and physical activity (yes: ≥ 1 times/week; no: never) were measured as close as possible to the DM diagnosis date.

### Statistical analyses

The characteristics of the study population were descriptively analyzed using the standardized mean difference (SMD). SMD values > 0.2 were regarded as potential imbalance between the two groups^40^. Conditional logistic regression analysis was conducted to investigate the association between metformin use and the risk of AD. We calculated the crude odds ratio (OR), adjusted OR (AOR), and 95% CI for the onset of AD between the metformin ever user and never-user groups. The analyses were adjusted for the following variables: hypertension, ischemic heart disease, dyslipidemia, CVD, chronic kidney disease, CCI, aDCSI, depression, fasting blood glucose levels, systolic blood pressure, diastolic blood pressure, total cholesterol levels, creatinine levels, statin use, cardiovascular medications (aspirin, statin, anticoagulant, antiplatelet, and antihypertension drugs), other antidiabetic medication, BMI, alcohol and smoking habits, and physical activity. Furthermore, we conducted subgroup analyses according to DM duration, and depression to investigate the heterogeneity of effect sizes. As sensitivity analyses, we conducted propensity score-matching to control covariate imbalance We used incident density random sampling for our primary analysis; however, the random imbalance of covariates between metformin user and never user may affect the main outcome. Therefore, we further conducted propensity score-matching (PS-matching) to control covariate imbalance. We conduct PS-matching using nearest neighbor matching method and baseline covariates in 1:5 ratio. A *p*-value < 0.05 was considered significant. All statistical analyses were performed using SAS software, version 9.4 (Cary, NC, USA).

## Supplementary Information

Supplementary Information.

## Data Availability

The data that support the findings of this study are NHIS-claims data and are stored on a separate server managed by the NHIS. The datasets generated and analyzed during the current study are not publicly available due to restrictions by NHIS. Access to the data is regulated by Korean law and the Korean National Institute for Health and Welfare. Interested parties may submit a separate application to the NHIS for access. The NHIS accepts applications via their website (https://nhiss.nhis.or.kr) and require ethics approval from the researcher’s institutional review board and a study proposal.
