# Supplementary material for: Association of metformin use with Alzheimer’s disease in patients with newly diagnosed type 2 diabetes: a population-based nested case–control study
Source: Sci Rep. 2021 Dec 15;11:24069. doi: 10.1038/s41598-021-03406-5 (PMC8674300; doi:10.1038/s41598-021-03406-5)
Supplement: Supplementary file 1 — Supplementary Information. [file 41598_2021_3406_MOESM1_ESM.docx]

**Supplementary Information**

**Association of metformin use with Alzheimer’s disease in patients with newly diagnosed type 2 diabetes: a population-based nested case-control study**

Junghee Ha, MD, PhD^1^†, Dong Woo Choi, PhD^2,3^†, Kwang Joon Kim MD, PhD ^4^, So Yeon Cho^1,5^, Hyunjeong Kim, PhD ^1^, Keun You Kim, MD^1^, Youngseung Koh^6^, Chung Mo Nam, PhD^7*^, Eosu Kim, MD, PhD^1,5*^

**Table e1. Risk of Alzheimer’s disease associated with metformin use stratified according to the duration of diabetes mellitus**

|  | **<5 years** | | | | | **5-9 years** | | | | | | **≥10 years** | | | | |
| --- | --- | --- | --- | --- | --- | --- | --- | --- | --- | --- | --- | --- | --- | --- | --- | --- |
|  | **Case** | **Controls** | | **AOR (95% Cl)** | | **Case** | | **Controls** | | **AOR (95% Cl)** | | **Case** | **Controls** | | **AOR (95% Cl)** | |
|  | **n (%)** | **n (%)** | |  |  | **n (%)** | | **n (%)** | |  |  | **n (%)** | **n (%)** | |  |  |
| Metformin use | | | | | | | | | | | | | | | | |
| Never user | 32 (16.5) | | 164 (17.5) | | 1 | | 78 (8.6) | | 558 (12.5) | | 1 | 24 (4.2) | | 255 (9.0) | | 1 |
| Ever user | 162 (83.5) | | 775 (82.5) | | 0.88 (0.54-1.43) | | 828 (91.4) | | 3897 (87.5) | | 1.48 (1.14-1.91) | 551 (95.8) | | 2582 (91.0) | | 2.18 (1.41-3.39) |
|  | | | | | | | | | | | | | | | | |
| Metformin (cDDD) | | | | | | | | | | | | | | | | |
| Never user | 32 (16.5) | | 164 (17.5) | | 1 | | 78 (8.6) | | 558 (12.5) | | 1 | 24 (4.2) | | 255 (9.0) | | 1 |
| Ever user |  | |  | |  | |  | |  | |  |  | |  | |  |
| Q1 | 42 (21.7) | | 196 (20.9) | | 0.82 (0.46-1.46) | | 224 (24.7) | | 958 (21.5) | | 1.66 (1.24-2.21) | 147 (25.6) | | 637 (22.5) | | 2.40 (1.51-3.82) |
| Q2 | 41 (21.1) | | 190 (20.2) | | 1.01 (0.57-1.80) | | 200 (22.1) | | 981 (22.0) | | 1.43 (1.06-1.92) | 138 (24.0) | | 645 (22.7) | | 2.11 (1.32-3.37) |
| Q3 | 45 (23.2) | | 189 (20.1) | | 1.04 (0.51-1.85) | | 216 (23.8) | | 966 (21.7) | | 1.49 (1.11-2.00) | 139 (24.2) | | 644 (22.7) | | 2.17 (1.36-3.49) |
| Q4 | 34 (17.5) | | 200 (21.3) | | 0.67 (0.36-1.24) | | 188 (20.8) | | 992 (22.3) | | 1.27 (0.94-1.72) | 127 (22.1) | | 656 (23.1) | | 1.98 (1.23-3.19) |
| Metformin (cDDD per day) | | | | | | | | | | | | | | | | |
| Never user | 32 (16.5) | | 164 (17.5) | | 1 | | 78 (8.6) | | 558 (12.5) | | 1 | 24 (4.2) | | 255 (9.0) | | 1 |
| Ever user |  | |  | |  | |  | |  | |  |  | |  | |  |
| Q1 | 48 (24.7) | | 288 (30.7) | | 0.79 (0.46-1.39) | | 239 (26.4) | | 1404 (31.5) | | 1.30 (0.98-1.73) | 158 (27.5) | | 790 (27.9) | | 2.15 (1.35-3.41) |
| Q2 | 26 (13.4) | | 107 (11.4) | | 0.99 (0.52-1.87) | | 140 (15.5) | | 580 (13.0) | | 1.59 (1.16-2.18) | 105 (18.3) | | 512 (18.1) | | 2.05 (1.27-3.32) |
| Q3 | 45 (23.2) | | 188 (20.0) | | 0.93 (0.51-1.67) | | 209 (23.1) | | 972 (21.8) | | 1.44 (1.07-1.93) | 143 (24.9) | | 642 (22.6) | | 2.24 (1.40-3.58) |
| Q4 | 43 (22.2) | | 192 (20.5) | | 0.89 (0.50-1.59) | | 240 (26.5) | | 941 (21.1) | | 1.70 (1.27-2.28) | 145 (25.2) | | 638 (22.5) | | 2.30 (1.43-3.68) |

cDDDs cumulative defined daily doses; AOR, adjusted odds ratio; CI, confidence interval

* Analysis was adjusted for the following covariates: hypertension, ischemic heart disease, dyslipidemia, Charlson comorbidity index, Diabetes complications severity index, depression, statin use, aspirin use, antiplatelet use, anticoagulant use, antihypertensive drug use, antiarrhythmic drug use, use of antidiabetic medications, fasting blood glucose levels, systolic blood pressure, diastolic blood pressure, total cholesterol levels, creatinine levels, body mass index, smoking status, alcohol consumption, and physical activity.

**Table e2. Comparison of the risk of Alzheimer’s disease associated with metformin use between patients with and without depression**

|  | **Without depression** | | |  | **With depression** | | |
| --- | --- | --- | --- | --- | --- | --- | --- |
|  | **Cases**  **(n = 1,213)** | **Controls**  **(n = 5,085)** | **AOR (95% Cl)** |  | **Cases**  **(n = 278)** | **Controls**  **(n = 415)** | **AOR (95% Cl)** |
|  |  |  |  |  |  |  |  |
|  | **n (%)** | **n (%)** |  |  | **n (%)** | **n (%)** |  |
| Metformin use | | | |  |  |  |  |
| Never user | 100 (8.2) | 645 (12.7) | 1 |  | 18 (6.5) | 39 (9.4) | 1 |
| Users | 1113 (91.8) | 4440 (87.3) | 1.57 (1.24-1.98) |  | 260 (93.5) | 376 (90.6) | 2.05 (1.02-4.12) |
| Metformin (cDDD) | | | |  |  |  |  |
| Never user | 100 (8.2) | 645 (12.7) |  | Never user | 18 (6.5) | 39 (9.4) |  |
| Ever user |  |  |  | Ever user |  |  |  |
| Q1 (<180cDDDs) | 294 (24.2) | 1099 (21.6) | 1.68 (1.31-2.17) | Q1 (<176.3 cDDDs) | 71 (25.5) | 88 (21.2) | 2.70 (1.24-5.89) |
| Q2 (180-495 cDDDs) | 265 (21.9) | 1119(22.0) | 1.54 (1.19-2.00) | Q2 (176.3-502.7 cDDDs) | 61 (21.9) | 98 (23.6) | 1.92 (0.87-4.24) |
| Q3 (495-1037.5 cDDDs) | 277 (22.8) | 1111 (21.9) | 1.50 (1.16-1.95) | Q3 (502.7-974.8 cDDDs) | 74 (26.6) | 85 (20.5) | 2.55 (1.17-5.55) |
| Q4 (≥1037.5 cDDDs) | 277 (22.8) | 1111 (21.9) | 1.50 (1.15-1.95) | Q4 (≥974.8 cDDDs) | 54 (19.4) | 105 (25.3) | 1.38 (0.80-3.01) |
| Metformin (cDDD per day) | |  |  |  |  |  |  |
| Never user | 100 (8.2) | 645 (12.7) |  |  | 18 (6.5) | 39 (9.4) |  |
| Ever user |  |  |  |  |  |  |  |
| Q1 (< 0.25 cDDDs/day) | 311 (25.6) | 1500 (29.5) | 1.41 (1.10-1.82) | Q1 (< 0.25 cDDDs/day) | 83 (29.9) | 150 (36.1) | 2.02 (0.95-4.28) |
| Q2 (0.25-0.32 cDDDs/day) | 199 (16.4) | 767 (15.1) | 1.56 (1.19-2.06) | Q2 (0.25-0.30 cDDDs/day) | 36 (13.0) | 49 (11.8) | 2.05 (0.87-4.84) |
| Q3 (0.32-0.47cDDDs/day) | 296 (24.4) | 1091 (21.5) | 1.65 (1.27-2.13) | Q3 (0.30-.44 cDDDs/day) | 77 (27.7) | 82 (19.8) | 2.88 (1.31-6.32) |
| Q4 (≥0.47 cDDDs/day) | 307 (25.3) | 1082 (21.3) | 1.71 (1.32-2.22) | Q4 (≥0.44cDDDs/day) | 64 (23.0) | 95 (22.9) | 1.71 (0.80-3.65) |

cDDDs cumulative defined daily doses; AOR, adjusted odds ratio; CI, confidence interval

* Analysis was adjusted for the following covariates: hypertension, ischemic heart disease, dyslipidemia, Charlson comorbidity index, Diabetes complications severity index, depression, statin use, aspirin use, antiplatelet use, anticoagulant use, antihypertensive drug use, antiarrhythmic drug use, use of antidiabetic medications, fasting blood glucose levels, systolic blood pressure, diastolic blood pressure, total cholesterol levels, creatinine levels, body mass index, smoking status, alcohol consumption, and physical activity.

| **Table e3. Characteristics of the study population stratified according to metformin use** | | | |
| --- | --- | --- | --- |
| **Variables** | **Never user**  **(n = 1,131)** | **Ever user**  **(n = 8,920)** | ***SMD*** |
|  | **n (%)** | **n (%)** |  |
| Age |  |  | 0.110 |
| < 75 years | 671 (59.4) | 5773 (59.4) |  |
| ≥ 75 years | 459 (40.6) | 3147 (35.3) |  |
| Women | 617 (54.6) | 5047 (56.6) | 0.040 |
| Diabetes duration |  |  | 0.275 |
| <5 years | 200 (17.7) | 963 (10.8) |  |
| 5-10 years | 649 (57.4) | 4781 (53.6) |  |
| ≥10 years | 281 (24.9) | 3176 (35.6) |  |
| BMI |  |  | 0.104 |
| < 18.5 kg/m^2^ | 9 (0.8) | 113 (1.3) |  |
| 18.5-22.9 kg/m^2^ | 277 (24.5) | 1921 (21.5) |  |
| 23-25 kg/m^2^ | 256 (22.7) | 2322 (26.0) |  |
| ≥ 25 kg/m^2^ | 588 (52.0) | 4564 (51.2) |  |
| Fasting blood glucose (mg/dL) † | 127.21 ± 40.99 | 134.81 ± 50.95 | 0.165 |
| BP (mmHg) † |  |  |  |
| Systolic | 136.42 ± 18.06 | 134.07 ± 17.37 | 0.132 |
| Diastolic | 81.11 ±10.98 | 80.76 ± 10.73 | 0.032 |
| Total cholesterol (mg/dL) † | 204.06 ± 41.61 | 204.48 ± 41.66 | 0.010 |
| Creatinine (mg/dL) † | 1.04 ± 0.91 | 0.99 ± 0.85 | 0.052 |
| Hypertension | 988 (87.4) | 7,704 (86.4) | 0.032 |
| Ischemic heart disease | 324 (28.7) | 2,685 (30.1) | 0.031 |
| Dyslipidemia | 615 (54.4) | 5,878 (65.9) | 0.236 |
| CCI |  |  | 0.126 |
| 0 | 448 (39.6) | 3017 (33.8) |  |
| 1 | 251 (22.2) | 2047 (22.9) |  |
| 2 | 431 (38.1) | 3856 (43.2) |  |
| aDCSI |  |  | 0.008 |
| 0 | 993 (87.9) | 7817 (87.6) |  |
| 1 | 96 (8.5) | 779 (8.7) |  |
| 2 | 41 (3.6) | 324 (3.6) |  |
| Depression | 176 (15.6) | 1681 (18.8) | 0.087 |
| Medication |  |  |  |
| Statin | 628 (55.6) | 6100 (68.4) | 0.266 |
| Aspirin | 685 (60.6) | 5867 (65.8) | 0.107 |
| Antiplatelet | 213 (18.8) | 1797 (20.1) | 0.033 |
| Anticoagulant | 45 (4.0) | 297 (3.3) | 0.035 |
| Antihypertensive agents | 929 (82.2) | 7124 (79.9) | 0.060 |
| Antiarrhythmic agents | 117 (10.4) | 1101 (12.3) | 0.063 |
| Smoking |  |  | 0.031 |
| None | 859 (76.0) | 6694 (75.0) |  |
| Past | 117 (10.4) | 913 (10.2) |  |
| Current | 154 (13.6) | 1313 (14.7) |  |
| Alcohol use |  |  | 0.032 |
| Low | 917 (81.2) | 7159 (80.3) |  |
| Moderate | 148 (13.1) | 1265 (14.2) |  |
| Heavy | 65 (5.8) | 496 (5.6) |  |
| Physical activity |  |  | 0.028 |
| Yes (≥1 time per week) | 784 (69.4) | 6075 (68.1) |  |

CCI, Charlson Comorbidity Index; aDCSI, adapted Diabetes Complication Severity Index; DPP-IV, dipeptidyl peptidase IV; SGLT-2, Sodium glucose cotransporter 2; SMD, Standardized mean difference.

^†^Mean and standard deviation (SD) of the continuous independent variables in this study.

**Table e4. Patients characteristic in PS matched cohort**

| **Variables** | **Before PS-matching** | | | **After PS-matching** | | |
| --- | --- | --- | --- | --- | --- | --- |
|  | **Cases  (n=2,056)** | **Controls  (n=91,207)** | **SMD** | **Cases  (n=2,027)** | **Controls  (n=9,708)** | **SMD** |
|  | **n (%)** | **n (%)** |  | **n (%)** | **n (%)** |  |
| BMI |  |  | 0.164 |  |  | 0.023 |
| < 18.5 kg/m^2^ | 36 (1.8) | 808 (0.9) |  | 33 (1.6) | 135 (1.4) |  |
| 18.5-22.9 kg/m^2^ | 524 (25.5) | 18445 (20.2) |  | 513 (25.3) | 2,410 (24.8) |  |
| 23-25 kg/m^2^ | 533 (25.9) | 23159 (25.4) |  | 528 (26.0) | 2,541 (26.2) |  |
| ≥ 25 kg/m^2^ | 963 (46.8) | 48795 (53.5) |  | 953 (47.0) | 4,622 (47.6) |  |
| Fasting blood glucose (mg/dL) † | 132.31 (50.7) | 131.3 (46.4) | 0.021 | 132.1 (50.8) | 132.0 (47.6) | 0.002 |
| BP (mmHg) † |  |  |  |  |  |  |
| Systolic | 133.3 (17.4) | 133.8 (17.5) | 0.031 | 133.3 (17.4) | 133.2 (17.3) | 0.001 |
| Diastolic | 80.3 (10.6) | 81.2 (10.9) | 0.080 | 80.3 (10.6) | 80.3 (10.7) | 0.003 |
| Total cholesterol (mg/dL) † | 203.8 (41.3) | 204.8 (41.6) | 0.025 | 203.8 (41.4) | 204.6 (42.1) | 0.021 |
| Creatinine (mg/dL) † | 0.99 (0.81) | 1.00 (0.89) | 0.015 | 0.99 (0.82) | 0.98 (0.79) | 0.005 |
| Depression | 535 (26.0) | 14,331 (15.7) | 0.256 | 527 (26.0) | 2,053 (21.1) | 0.114 |
| Ischemic heart disease | 629 (30.6) | 26,962 (29.6) | 0.023 | 621 (30.6) | 2,992 (30.8) | 0.004 |
| Dyslipidemia | 1,268 (61.7) | 60,153 (66.0) | 0.089 | 1,256 (62.0) | 6,029 (62.1) | 0.003 |
| CCI |  |  | 0.053 |  |  | 0.023 |
| 0 | 660 (32.1) | 29,838 (32.7) |  | 652 (32.2) | 3,111 (32.0) |  |
| 1 | 516 (25.1) | 20,859 (22.9) |  | 504 (24.9) | 2,332 (24.0) |  |
| 2 | 880 (42.8) | 40,510 (44.4) |  | 871 (43.0) | 4,265 (43.9) |  |
| aDCSI |  |  | 0.100 |  |  | 0.047 |
| 0 | 1,759 (85.6) | 80,851 (88.6) |  | 1,736 (85.6) | 8,452 (87.1) |  |
| 1 | 223 (10.8) | 7,281 (8.0) |  | 219 (10.8) | 913 (9.4) |  |
| 2 | 74 (3.6) | 3,075 (3.4) |  | 72 (3.6) | 343 (3.5) |  |
| Medication |  |  |  |  |  |  |
| Statin | 1,305 (63.5) | 59,217 (64.9) | 0.030 | 1,286 (63.4) | 6,134 (63.2) | 0.005 |
| Aspirin | 1,311 (63.8) | 57,920 (63.5) | 0.005 | 1,290 (63.6) | 6,136 (63.2) | 0.009 |
| Antiplatelet | 482 (23.4) | 15,953 (17.5) | 0.148 | 474 (23.4) | 2,124 (21.9) | 0.036 |
| Anticoagulant | 96 (4.7) | 2,551 (2.8) | 0.099 | 95 (4.7) | 409 (4.2) | 0.023 |
| Antihypertensive agents | 1,676 (81.5) | 70,597 (77.4) | 0.102 | 1,653 (81.5) | 7,882 (81.2) | 0.009 |
| Antiarrhythmic agents | 288 (14.0) | 10,744 (11.8) | 0.067 | 285 (14.1) | 1,300 (13.4) | 0.019 |
| Alcohol use |  |  | 0.107 |  |  | 0.004 |
| Low | 1,626 (79.1) | 71,081 (77.9) |  | 1,603 (79.1) | 7,662 (78.9) |  |
| Moderate | 283 (13.8) | 15,278 (16.8) |  | 280 (13.8) | 1,348 (13.9) |  |
| Heavy | 147 (7.1) | 4,848 (5.3) |  | 144 (7.1) | 698 (7.2) |  |
| Smoking |  |  | 0.039 |  |  | 0.002 |
| None | 1,523 (74.1) | 66,778 (73.2) |  | 1,502 (74.1) | 7,194 (74.1) |  |
| Past | 202 (9.8) | 10,049 (11.0) |  | 198 (9.8) | 943 (9.7) |  |
| Current | 331 (16.1) | 14,380 (15.8) |  | 327 (16.1) | 1,571 (16.2) |  |
| Physical activity |  |  |  |  |  |  |
| Yes (≥1 time per week) | 1,295 (63.0) | 64,457 (70.7) | 0.164 | 1,277 (63.0) | 6,285 (64.7) | 0.036 |
| Antidiabetic medication |  |  |  |  |  |  |
| Alpha-glucosidase inhibitors | 192 (9.3) | 8,172 (9.0) | 0.013 | 190 (9.4) | 874 (9.0) | 0.013 |
| DPP-IV inhibitors | 622 (30.3) | 23,778 (26.1) | 0.093 | 615 (30.3) | 2,780 (28.6) | 0.037 |
| Insulin | 792 (38.5) | 25,897 (28.4) | 0.216 | 785 (38.7) | 3,515 (36.2) | 0.052 |
| SGLT-2 inhibitors | 13 (0.6) | 839 (0.9) | 0.033 | 13 (0.6) | 65 (0.7) | 0.003 |
| Sulfonylurea | 1556 (75.7) | 68,054 (74.6) | 0.025 | 1,540 (76.0) | 7,382 (76.0) | 0.002 |
| Thiazolidinedione | 230 (11.2) | 12,674 (13.9) | 0.082 | 229 (11.3) | 1,136 (11.7) | 0.013 |

**Table e5. Risk of Alzheimer’s disease associated with metformin use in diabetes mellitus patients after PS-matching**

|  | **Cases  (n=2,027)** | **Controls  (n=9,708)** | **AOR (95% Cl)** |
| --- | --- | --- | --- |
|  | **n (%)** | **n (%)** |  |
| Metformin use |  |  |  |
| Never user | 333 (16.4) | 1,550 (16.0) | 1.00 |
| Users | 1,694 (83.6) | 8,158 (84.0) | 1.25 (1.07-1.44) |
| Cumulative dose of use |  |  |  |
| Never user | 457 (22.5) | 2,007 (20.7) | 1.00 |
| Ever user |  |  |  |
| Q1 (<131cDDDs) | 427 (21.1) | 2,035 (21.0) | 1.37 (1.15-1.62) |
| Q2 (131-447 cDDDs) | 413 (20.4) | 2,050 (21.1) | 1.23 (1.03-1.46) |
| Q3 (448-966 cDDDs) | 397 (19.6) | 2,066 (21.3) | 1.18 (0.98-1.40) |
| Q4 (≥966 cDDDs) | 333 (16.4) | 1,550 (16.0) | 1.13 (0.94-1.36) |
| Cumulative dose of use per day |  |  |  |
| Never user | 543 (26.8) | 3,029 (31.2) | 1.00 |
| Ever user |  |  |  |
| Q1 (< 0.25 cDDDs/day) | 242 (11.9) | 1,113 (11.5) | 1.10 (0.93-1.29) |
| Q2 (0.25-0.31 cDDDs/day) | 438 (21.6) | 2,024 (20.8) | 1.32 (1.08-1.61) |
| Q3 (0.32-0.46 cDDDs/day) | 471 (23.2) | 1,992 (20.5) | 1.31 (1.10-1.57) |
| Q4 (≥0.46 cDDDs/day) | 333 (16.4) | 1,550 (16.0) | 1.46 (1.22-1.74) |

**Figure e1. Odds ratios for Alzheimer’s disease in different subgroups in overall population**

Adjusted odds ratios with 95% confidence intervals for multivariable models evaluating associations between metformin use and incident Alzheimer’s disease according to subgroup. Boxes indicate the odds ratio, limit lines indicate the 95% confidence interval, and the horizontal indicates odds ratio. Metformin never users were considered as the reference.
